# Electrospun Gelatin Fibers Surface Loaded ZnO Particles as a Potential Biodegradable Antibacterial Wound Dressing

**DOI:** 10.3390/nano9040525

**Published:** 2019-04-03

**Authors:** Yu Chen, Weipeng Lu, Yanchuan Guo, Yi Zhu, Yeping Song

**Affiliations:** 1 Key Laboratory of Photochemical Conversion and Optoelectronic Material, Technical Institute of Physics and Chemistry, Chinese Academy of Sciences, Beijing 100190, China; chenyubrc@mail.ipc.ac.cn; 2 University of Chinese Academy of Sciences, Beijing 100049, China; 3 Hangzhou Research Institute of Technical Institute of Physics and Chemistry, Chinese Academy of Sciences, Hangzhou 310018, China; zhuyi@mail.ipc.ac.cn (Y.Z.); songyeping@mail.ipc.ac.cn (Y.S.)

**Keywords:** gelatin fibers, ZnO particles, antibacterial activity

## Abstract

Traditional wound dressings require frequent replacement, are prone to bacterial growth and cause a lot of environmental pollution. Therefore, biodegradable and antibacterial dressings are eagerly desired. In this paper, gelatin/ZnO fibers were first prepared by side-by-side electrospinning for potential wound dressing materials. The morphology, composition, cytotoxicity and antibacterial activity were characterized by using Fourier transform infrared spectroscopy (FTIR), X-ray diffractometry (XRD), particle size analyzer (DLS), scanning electron microscopy (SEM), energy dispersive X-ray spectroscopy (EDX), thermogravimetry (TGA) and Incucyte™ Zoom system. The results show that ZnO particles are uniformly dispersed on the surface of gelatin fibers and have no cytotoxicity. In addition, the gelatin/ZnO fibers exhibit excellent antibacterial activity against *Staphylococcus aureus* (*S. aureus*) and *Escherichia coli* (*E. coli*) with a significant reduction of bacteria to more than 90%. Therefore, such a biodegradable, nontoxic and antibacterial fiber has excellent application prospects in wound dressing.

## 1. Introduction

Skin, as the human body’s largest organ, exerts a vital role in protecting the human body from external harm. Skin damage can lead to microbial invasion of the human body, resulting in a threat to human health [1,2,3]. Medical dressing is a kind of medical equipment which can cover damaged skin and form a microenvironment conducive to wound healing, thus playing an effective role in wound care and treatment. At present, most medical dressings still use traditional cotton gauze. Nevertheless, traditional cotton gauze needs to be replaced frequently, and easily adheres to the wound, which can easily lead to secondary tissue trauma and bacterial breeding [4,5,6]. Moreover, huge dressing wastes cause great harm to the environment. Therefore, it is urgent to design new biodegradable and biocompatible dressing materials with good antimicrobial activity.

Gelatin, as a kind of natural material, is hydrolyzed from collagen in animals, which has many advantages, such as good accessibility, a wide source of raw materials and low cost. Its amino acid composition is similar to collagen and has good biocompatibility, biodegradability and low immunogenicity [7,8,9]. Especially in the past decades, with the development of electrospinning technology, gelatin fibers can be prepared simply and quickly. Furthermore, there are many small secondary structures on the surface of gelatin fibers, which is similar to the structure of extracellular matrix (ECM) and closer to the structural size of organisms. Therefore, it plays an important part in cell attachment, growth, migration and differentiation, as well as the formation of new tissues [10,11,12]. Meanwhile, gelatin fibers have strong adsorption, good filtration, barriers, adhesion and hygroscopicity [13,14,15,16]. Therefore, the gelatin 3D nanostructures prepared by electrospinning can be widely used in the biomedical materials, which receives more and more attention. In addition, in recent years nano-inorganic ions and nano-metal oxides have been found to have extensive antibacterial properties, which caused widespread concern [17,18,19]. Among them, ZnO particles were generally recognized as a safe (GRAS) material by the Food and Drug Administration (FDA) [20]. It has excellent antibacterial properties and minimal effect on human cells, which is extensively used in biomedicine and health products [21,22,23].

Recently, the blending of ZnO particles with gelatin has been used in the research of antimicrobial materials. Liu et al. prepared gelatin/ethyl cellulose/ZnO nanofibers by electrospinning. The results showed that the nanofibers had a good inhibitory effect on *Escherichia coli* (*E. coli*) and *Staphylococcus aureus* (*S. aureus*) [24]. Chhabra et al. synthesized ZnO doped gelatin and poly-methyl vinyl ether-*alt*-maleic anhydride (PMVE/MA) composite electrospun scaffolds, which can inhibit bacterial activity and have no cytotoxicity to mammalian cells [25]. Münchow et al. prepared ZnO loaded gelatin/polycaprolactone (PCL) composite nanofibers by electrospinning technique [26]. At present, the current preparation of ZnO/gelatin fibers is usually carried out in the form of blending spinning. Nevertheless, ZnO particles are hard to fully exert efficient antibacterial activity when encapsulated in gelatin fibers. Consequently, it is necessary to increase the content of ZnO particles to achieve better antibacterial effects [27]. However, with that, excess ZnO particles cause waste of materials, and even more seriously excess ZnO particles may lead to cytotoxic effects and affect the growth of the tissue [28,29,30].

**Figure 1:**
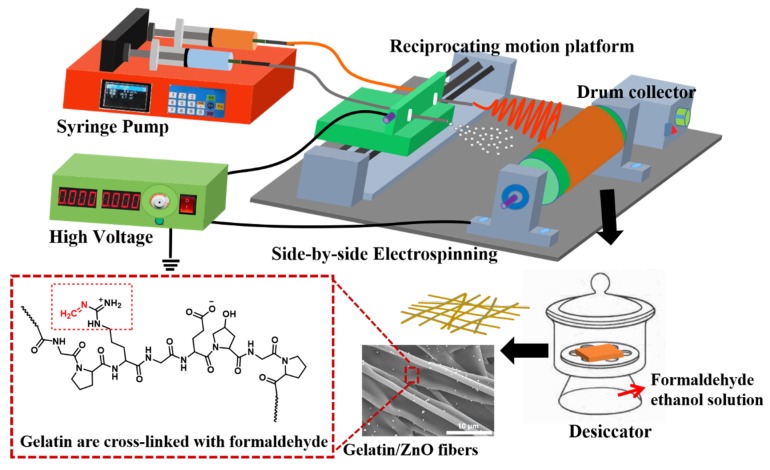
Schematic illustration of the fabrication of gelatin/ZnO fibers by side-by-side electrospinning.

In this paper, unlike the traditional blending method, ZnO particles were dispersed in ethanol, so that ZnO particles can follow the solvent to be sprayed on gelatin fibers by using the side-by-side spray nozzles in the electrospinning process. With the volatilization of ethanol solvent, ZnO particles can be uniform spread only on the surface of gelatin fibers (as shown in Figure 1), so as to achieve the best antibacterial effect with the minimum content of ZnO particles.

## 2. Materials and Methods

### 2.1. Materials

Gelatin (type B, basic-processed, prepared by bones, with a molecular weight of 100,000, viscosity value of 4.9 MPa·s^−1^) was obtained from Dongbao Bio-Tech Co., Ltd. (Baotou, China). 2,2,2-trifluoroethanol, formaldehyde solution (37–40 wt %), zinc acetate dihydrate (Zn(CH_3_COO)_2_·2H_2_O), diethylene glycol and ethanol absolute were purchased from Titan Scientific Co., Ltd. (Shanghai, China). The human lung fibroblast cell lines (MRC-5) were purchased from the Cell Bank of Chinese Academy of Sciences (Shanghai, China). *Staphylococcus aureus* (*S. aureus*) and *Escherichia coli* (*E. coli*) were obtained from the China General Microbiological Culture Collection Center (Beijing, China). The deionized water had a resistivity of 18.2 MΩ·cm in the process of the experiments. All chemical reagents were of analytical grade and required no post-treatment.

### 2.2. Preparation of ZnO Particles

For preparation of ZnO particles [31], 878 mg (0.004 mol) Zn(CH_3_COO)_2_·2H_2_O was added into 40 mL diethylene glycol with continued ultrasonic until dissolved completely, followed by transferring into a teflon-lined stainless steel autoclave (45 mL). Then, the autoclave was heated to 160 °C from room temperature in an oven and maintained for 5 h. After the reaction, the white power was obtained by centrifugation. Then, the power was washed with ethanol and water in turn, and finally dried in the oven at 100 °C for 5 h.

### 2.3. Preparation of Gelatin/ZnO Fibers

For preparation of gelatin/ZnO fibers, gelatin (12.5%, w/v) was added into 2,2,2-trifluoroethanol with constant stirring at 45 °C until it was completely dissolved. Then, ZnO particles (0%, 0.10%, 0.25%, respectively) were evenly dispersed in the ethanol solution by ultrasonic. In the electrospinning process, gelatin solution and ZnO particles solution were added into 50 mL syringes respectively, in which the diameter of the syringe needle containing gelatin was 1.25 mm and that containing ZnO particles was 0.80 mm. The distance from the drum collector to the needle tip was 15 cm, and the applied voltage was 17 kV. The gelatin fibers and ZnO particles were spun with a flow rate of 5 and 2 mL·h^−1^, respectively. In addition, the speed of reciprocating motion platform and drum collector were 55 and 70 r·min^−1^, respectively. After electrospinning, the fibers were placed on the porous ceramic shelf of the desiccator, and the ethanol solution containing 1% formaldehyde solution was added at the bottom. Then, the desiccator was put into the oven at 25 °C for 48 h. After that, the cross-linked fibers were put into the oven at 40 °C for 24 h to remove excess formaldehyde. GZ0’, GZ1’ and GZ2’ represent the gelatin/ZnO fibers, in which the concentration of ZnO particles solution is 0%, 0.1% and 0.25% in the electrospinning process, respectively. GZ0, GZ1 and GZ2 represent GZ0’, GZ1’ and GZ2’ after cross-linking.

### 2.4. Characterization

The microstructure and chemical composition of the gelatin/ZnO fibers were characterized by a S-4300 scanning electron microscope (SEM, Hitachi, Tokyo, Japan) and energy dispersive X-ray spectroscopy (EDX, Hitachi, Tokyo, Japan). The size of ZnO particles was analyzed by the dynamic light scattering (DLS) method (litesizer^TM^ 500, Anton Paar GmbH, Graz, Austria). Samples were dispersed in the ethanol, the concentration of the sample was 1 mg·mL^−1^, and the pH value was 6.5. The functional groups in the fibers were characterized by Fourier transform infrared spectroscopy (FTIR) (Bruker Tensor II, Karlsruhe, Germany), and the scanning range of the samples was 4000–400 cm^−1^. The crystal structures of the samples were determined by X-ray diffraction (XRD, ARL XTRA, Zurich, Switzerland). The scanning rate was 0.1 s·step^−1^ and the scanning range was 10–80°. The thermal stability of the samples was analyzed by thermogravimetry (TGA, NETZSCH, Selb, Germany) in nitrogen atmosphere, and the heating rate was 10 °C·min^−1^. The Incucyte^TM^ Zoom system (EssenBio, Ann Arbor, MI, USA) was used to observe effect of the extraction on MRC-5 cells in real time. The extraction and 10% FBS were placed in 96-well plates. Each well was inoculated with 2000 cells and cultured in an atmosphere of 5% CO_2_ at 37 °C for seven days. Phase contrast images were acquired every 3 h.

### 2.5. Stability of GZ2 in Aqueous Solutions

The stability of cross-linked fibers in aqueous solution was studied by taking GZ2 as an example. Specifically, GZ2 was cut into a 2 × 0.5 × 0.2 cm^3^ shape (20 mg) and placed in a bottle containing 10 mL phosphate buffer saline (PBS, 0.01 M, pH = 7.2–7.4). Then, the bottles were placed in the oven at 37 °C and recorded every 24 h. The experiment was done in triplicate.

### 2.6. Cell Culture and Proliferation

MRC-5 cells were used to assess the cell culture and proliferation. The cells were cultured in a humidified chamber containing 5% CO_2_ at 37 °C using Dulbecco’s modified eagle’s medium/F-12 (DME/F-12) containing 10% fetal bovine serum. Extraction experiment was carried out according to the instruction of ISO 10993-12: 2002. Serum free cell culture medium (DME/F-12) was used to obtain the extraction media of the GZ0, GZ1 and GZ2. The extraction ratio (the ratio of sample quality to extraction medium) was 0.1 g·mL^−1^. GZ0, GZ1 and GZ2 soaked in the medium were incubated in a humidified atmosphere with 5% CO_2_ at 37 °C for 24 h. Cell culture medium (DME/F-12) was used as a negative control.

### 2.7. Antibacterial Evaluation

*E. coli* and *S. aureus* were chosen to explore the antibacterial properties of gelatin/ZnO fibers by using the viable colony count method. Tryptic Soy Broth (TSB) and Trypticase Soy Agar (TSA) were used as culturing nutrient sources. Specifically, *E. coli* and *S. aureus* were aseptically inoculated in TSB and then cultured in a shaker at 37 °C for 16 h. Each of the cultured broths were continually cultured in new TBS in a shaker at 37 °C for another 3 h, and then each of the cultured broths were centrifuged and washed twice with sterile PBS (0.01 M, pH = 7.2–7.4). Finally, the bacterial PBS suspension with the concentration of 1 × 10^6^ colony forming units per milliliter (CFU·mL^−1^) was obtained by the gradient dispersion method. The sterile gelatin/ZnO fibers (GZ0, GZ1 and GZ2) were cut into the shape of 2 × 2 cm^2^ (30 mg) and then placed into tube containing 10 mL sterile PBS. One group of tubes was irradiated for 1 h under ultraviolet light (UV, 365 nm, 50 w), the other group was not irradiated. After the pretreatment, 0.1 mL of each bacterial suspension was added, and incubated in the shaker at 37 °C for 3 h. After that, a 10 μL solution was taken and serially diluted in sterile PBS. Then, 30 μL of each diluent was taken and spread onto a TSA plate, and then all plates were incubated for 16 h at 37 °C. The numbers of the suitable colonies that formed were counted. In addition, medium with only inoculum was used as negative control, and pure ZnO particles (30 mg) were used as positive control. All experiments were performed in triplicate.

### 2.8. Statistical Analysis

The data were expressed as mean ± standard deviation. Statistically significant differences of the samples were assessed using a Student’s *t*-test. *p* < 0.05 was considered to be statistically significant.

## 3. Results and Discussion

### 3.1. Morphologies of Gelatin/ZnO Fibers

#### 3.1.1. Characterization of ZnO Particles

**Figure 2:**
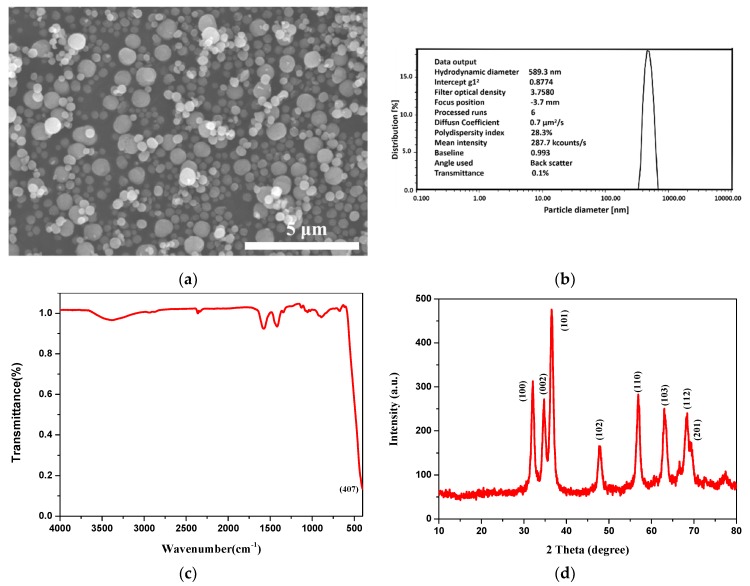
Characterization of ZnO particles (**a**) scanning electron microscopy (SEM) image, (**b**) particle size distribution by intensity, (**c**) Fourier transform infrared spectroscopy (FTIR) spectrum and (**d**) X-ray diffractometry (XRD) spectrum.

The characterization data of the ZnO particles are shown in Figure 2. Specifically, Figure 2a represents the SEM images of ZnO particles. It indicates that the ZnO particles have a regular spherical shape and are uniformly dispersed without agglomeration. The DLS measurements show that average hydrodynamic diameter is about 589.3 nm (Figure 2b). Figure 2c illustrates the FTIR spectrum of the ZnO particles. The single peak at 407 cm^−1^ can be assigned to stretching mode of the Zn-O bond [32]. To further determine the composition of the resulting particles, XRD was carried out. As shown in Figure 2d, the diffraction peaks at 2θ values of 31.8°, 34.5°, 36.3°, 47.8°, 56.3°, 63.2°, 68.0° and 69.2° correspond to (100), (002), (101), (102), (110), (103), (112) and (201) crystal planes of ZnO, respectively [33].

#### 3.1.2. Characterization of Gelatin/ZnO Fibers

**Figure 3:**
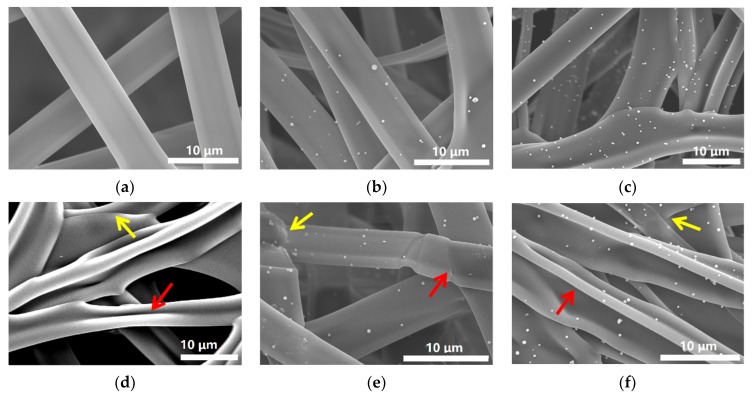
SEM images of (**a**) GZ0’, (**b**) GZ1’, (**c**) GZ2’, (**d**) GZ0, (**e**) GZ1 and (**f**) GZ2.

**Figure 4:**
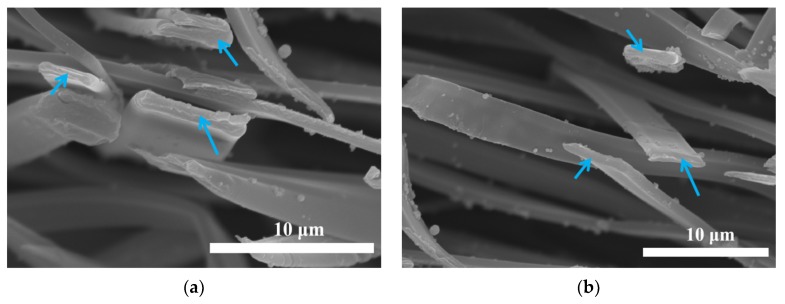
The cross sections SEM images of (**a**) GZ1’, (**b**) GZ2’.

**Figure 5:**
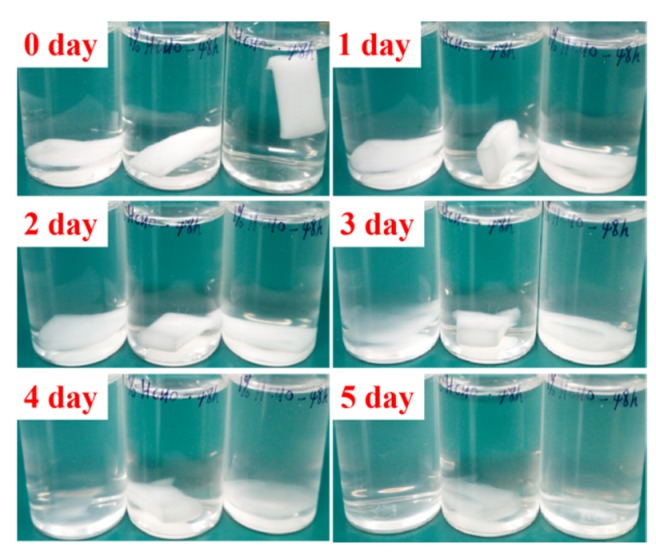
Degradation process diagram of GZ2 in phosphate buffer saline (PBS) at 37 °C.

SEM measurements were performed to study morphology of the fibers before and after crosslinking. As shown in Figure 3a–c, GZ0’, GZ1’ and GZ2’ represent the gelatin/ZnO fibers, in which the concentration of ZnO particles solution is 0%, 0.1% and 0.25% in the electrospinning process, respectively. The average diameters of GZ0’, GZ1’ and GZ2’ are about 6.22, 5.67 and 7.32 μM. The pure gelatin fiber (GZ0’) exhibits a uniform smooth surface, and the fiber thickness is consistent. With the addition of ZnO particles in electrospinning process, ZnO particles can be evenly dispersed on the surface of gelatin fibers (Figure 3b,c), but no particles are found inside GZ1’ and GZ2’ (indicated by blue arrow in Figure 4). The morphology of the fibers remains basically unchanged. Furthermore, as a potential wound dressing, gelatin/ZnO fibers need to have good physical and chemical stability in aqueous solution. Thus, formaldehyde was chosen as the cross-linking agent. GZ0, GZ1 and GZ2 (Figure 3d–f) represent the GZ0’, GZ1’ and GZ2’ after crosslinking, respectively. Figure 5 shows the degradation process of GZ2 in PBS at 37 °C. It can be seen that the degradation of GZ2 is basically completed after five days, which indicates that GZ2 has good water resistance. In addition, Figure 3d–e shows that the average diameter of the nanofibers increases after the cross-linking, and the fibers are curly (indicated by red arrow above). Meanwhile, the fibers become denser and fuse at some intersection points (shown by yellow arrow above).

**Figure 6:**
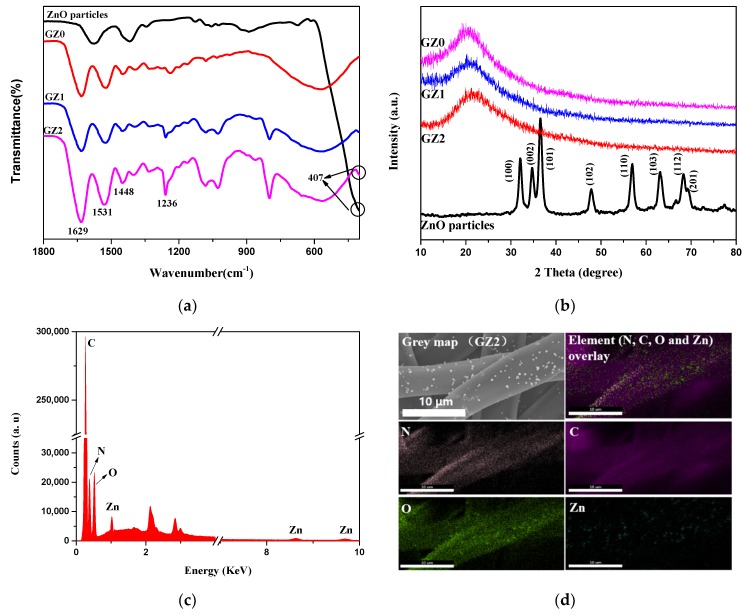
(**a**) FTIR spectra of ZnO particles, GZ0, GZ1 and GZ2, (**b**) XRD patterns of ZnO particles, GZ0, GZ1 and GZ2, (**c**) energy dispersive X-ray spectroscopy (EDX) analysis of GZ2, and (**d**) EDX mapping of GZ2.

To ascertain the chemical structures of gelatin/ZnO fibers, FTIR was first tested. As shown in Figure 6a, the spectra of GZ1 and GZ2 represent three characteristic peaks at 1629, 1531 and 1236 cm^−1^ corresponding to amide I, amide II and amide III, respectively. The amide I band is mainly attributed to the tensile vibration of -C=O, and the amide II and III bands are caused by the bending vibration of -NH and the stretching vibration of -C-N, respectively [34]. A weak absorption peak at 407 cm^−1^ in the GZ1 and GZ2 spectra is consistent with the characteristic absorption peak of ZnO (as mentioned in Section 3.1.1), which belongs to the stretching vibration of the Zn-O bond. In addition, due to the reaction between the aldehyde group of formaldehyde and the amino lysine residue of gelatin, the stretching vibration peak of the imide group (-CH=N) appears at 1448 cm^−1^ [35]. Furthermore, Figure 6b shows the XRD spectra of gelatin/Zn fibers as well as the ZnO particles. GZ0 has a broad diffraction peak at 2θ = 20° [36]. Unfortunately, no ZnO peaks are found in the GZ1 and GZ2 spectra, which may be due to the low content of ZnO particles on the surface of the gelatin [24]. In order to determine the types of elements contained in gelatin/ZnO fibers, EDX was carried out. The selected area for mapping is a part of GZ2, in which the ZnO particles are evenly loaded on the surface. Figure 6c,d represent EDX analysis and element mapping of GZ2, respectively. GZ2 shows the presence of C, N, O and Zn elements. The appearance of C and N elements is due to the presence of these two elements in gelatin molecules. The Zn element is caused by the presence of the element in ZnO particles. In addition, the O element exists both in gelatin molecules and ZnO particles. Moreover, Zn mapping of GZ2 verifies that ZnO particles are evenly dispersed on the surface of gelatin fibers.

**Figure 7:**
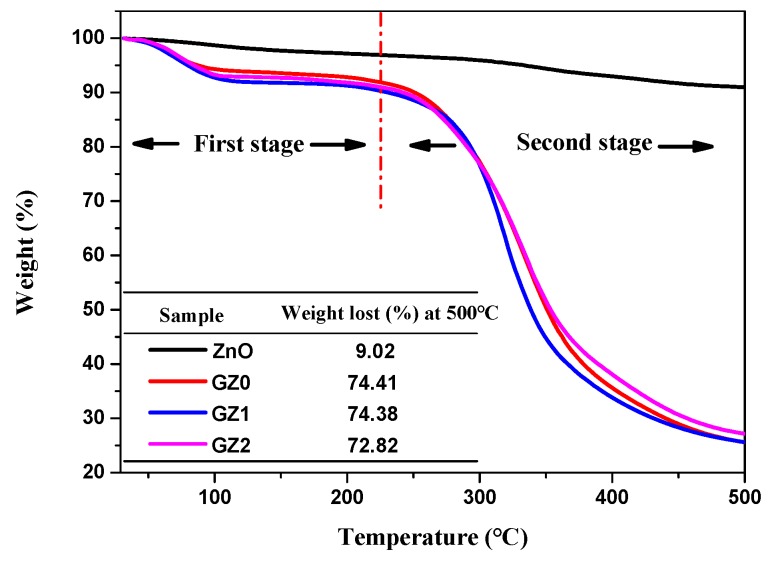
Thermogravimetric analysis (TGA) curve of ZnO, GZ0, GZ1 and GZ2.

TGA was carried out to study the thermal stability of gelatin/ZnO fibers. As shown in Figure 7, TGA curve of ZnO particles shows 9.02% weight lost at 500 °C, caused by the evaporation of physically adsorbed water and decomposition of residual acetate. Meanwhile, the TGA curves of GZ0, GZ1 and GZ2 are basically consistent, and the thermal decomposition process is divided into two stages [37,38]. In the first stage (30–224 °C), the weight lost is approximately 8.97%, which is caused by the evaporation of physically adsorbed water. The second stage is related to the decomposition of gelatin, and with the increase of ZnO content, the weight loss decreases gradually at the end of the decomposition process (500 °C). Obviously, the presence of ZnO has little effect on the thermal decomposition process of gelatin fibers, and the gelatin/ZnO fibers have good thermal stability below 224 °C.

### 3.2. Bioactivity Studies of Gelatin/ZnO Fibers

#### 3.2.1. Cell Activity

**Figure 8:**
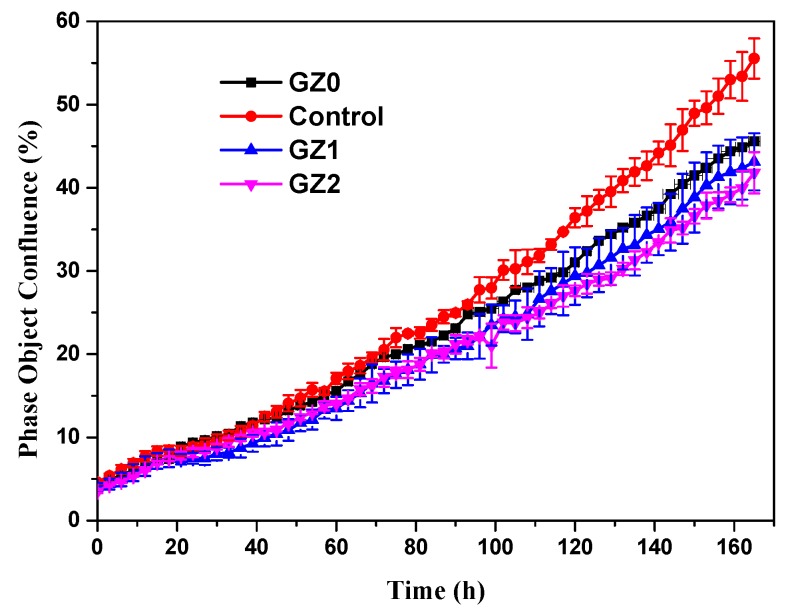
Real-time cell confluence study MRC-5 cells. The cell population was monitored for 168 h using an Incucyte™ Zoom system in an incubator (5% CO_2_ and 37 °C).

**Figure 9:**
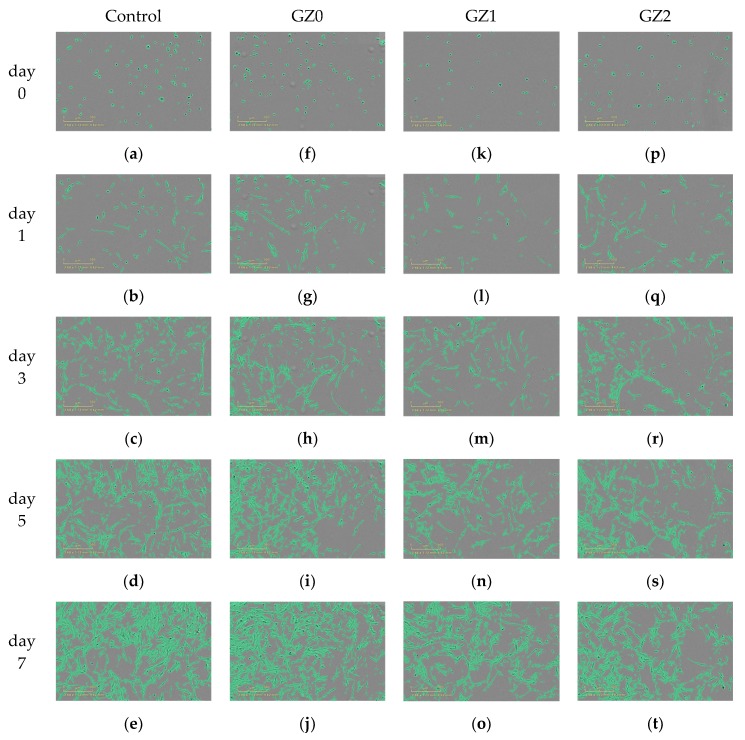
Cell morphology of (**a**–**e**) Control, (**f**–**j**) GZ0, (**k**–**o**) GZ1 and (**p**–**t**) GZ2 at point of day 0, 1, 3, 5 and 7, respectively. Scale bar: 500 μm.

The proliferation of GZ0, GZ1 and GZ2 were analyzed by real-time monitoring of MRC-5 cells using an Incucyte^TM^ Zoom microscope. In Figure 8, time-lapse imaging of the control exhibits a standard growth curve up to 55.5% cell confluence level by day 7, and the GZ0, GZ1 and GZ2 reach 45.6, 43.1 and 41.8% cell confluence level by day 7, respectively. The growth curve trends for GZ0, GZ1 and GZ2 are basically the same, and lower than that of the control. The results indicate that the GZ0, GZ1 and GZ2 have certain inhibiting effects on cell proliferation. Relative growth rate (RGR, %) was used to express the cytotoxicity. Table 1 shows that all the relative growth rates are more than 75%, corresponding to the cytotoxicity level of 0 or 1 per the standard in Table 2. This indicates that the gelatin/ZnO fibers (GZ0, GZ1 and GZ2) have no cytotoxicity. In addition, as shown in Figure 9, MRC-5 cells cultured in extraction from GZ0, GZ1 and GZ2 are marked by a green phase object mask via the software of Incucyte^TM^ Zoom. It can be seen that the cells display healthy spindle-like or star-like shape, and the number of cells rose with the extension of culture time, which suggests that GZ1 and GZ2 impose no suppression on the growth of cells.

#### 3.2.2. Antibacterial Activity

**Figure 10:**
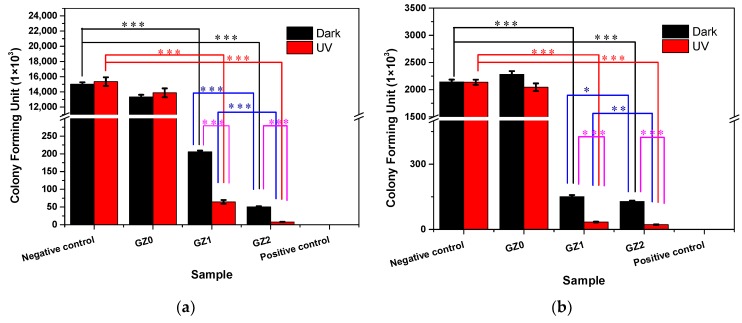
The antibacterial activity of the gelatin/ZnO fibers against (**a**) *Staphylococcus aureus* and (**b**) *Escherichia coli* with and without UV light. (* represents *p* < 0.05, ** represents *p* < 0.01 and *** represents *p* < 0.001.).

**Figure 11:**
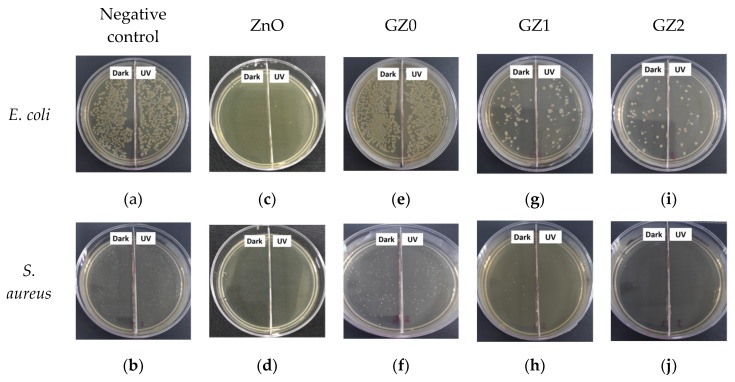
The photographs showing the antibacterial activity of (**a**,**b**) negative control, (**c**,**d**) ZnO particles, (**e**,**f**) GZ0, (**g**,**h**) GZ1 and (**i**,**j**) GZ2 against *S. aureus* and *E. coli*, respectively.

Figure 10 illustrates the antibacterial activity of the gelatin/ZnO fibers against *E. coli* and *S. aureus* with and without UV light. The bacteria were determined by using the viable colony count method. Figure 11 represents photographs showing the antibacterial activity of gelatin/ZnO fibers against *S. aureus* and *E. coli*. Obviously, pure gelatin fibers (GZ0) have no toxic effect on the bacterial strains, and pure ZnO, GZ1 and GZ2 have excellent bacteriostatic effects. In addition, GZ2 containing a higher concentration of ZnO has stronger antibacterial activity than GZ1 containing a low concentration of ZnO. According to the reported literature [39,40,41], the reason for the antibacterial activity of gelatin/ZnO fibers is the superoxide radicals (·O_2_^−^) produced by ZnO particles. The superoxide radicals can attack the bacterial cell wall and lead to cell wall leakage, resulting in the death of bacteria. In addition, under UV irradiation, ZnO particles can produce a large number of superoxide radicals. The obtained data (Figure 10) show that the antibacterial effect of gelatin/ZnO fibers irradiated by UV light is stronger than that of non-irradiated. This also proves the antibacterial mechanism of gelatin/ZnO fibers.

## 4. Conclusions

In summary, we first fabricated gelatin/ZnO fibers via side-by-side electrospinning technique as a potential wound dressing. The data indicate that ZnO particles can be uniformly dispersed on the surface of gelatin fibers. Although gelatin/ZnO fibers have some inhibitory effect on MRC-5 cells, the corresponding cytotoxicity level is 0 or 1 per the standard, which indicates that fibers have no cytotoxicity. Moreover, the gelatin/ZnO fibers show excellent antibacterial activity against *E. coli* and *S. aureus*, which is caused by the superoxide radicals (·O_2_^−^) produced by ZnO particles. In addition, the reductions of bacteria are all more than 90%. The experiments imply that gelatin/ZnO fibers have excellent application prospects for wound dressing.

## Figures and Tables

**Table 1:** The relative growth rate (RGR) and cytotoxicity level of GZ0, GZ1 and GZ1.

		Day 1	Day 3	Day 5	Day 7
**GZ0**	**RGR (%)**	105.8	95.7	85.2	82.1
	**Cytotoxicity level**	0	1	1	1
**GZ1**	**RGR (%)**	84.6	81.2	80.7	77.6
	**Cytotoxicity level**	1	1	1	1
**GZ2**	**RGR (%)**	92.4	84.1	75.8	75.3
	**Cytotoxicity level**	1	1	1	1

**Table 2:** The standard of cytotoxicity determined from RGR.

Cytotoxicity Level	0	1	2	3	4	5
**RGR (%)**	>100	75–99	50–74	25–49	1–24	0
